# Characterization of ML-005, a Novel Metaproteomics-Derived Esterase

**DOI:** 10.3389/fmicb.2018.01925

**Published:** 2018-08-22

**Authors:** Premankur Sukul, Natalie Lupilov, Lars I. Leichert

**Affiliations:** Department of Microbial Biochemistry, Institute of Biochemistry and Pathobiochemistry, Ruhr University Bochum, Bochum, Germany

**Keywords:** esterase, lipase, metagenomics, metaproteomics, biocatalysis

## Abstract

A novel gene encoding for a lipolytic enzyme, designated ML-005, was recently identified using a functional metaproteomics approach. We heterologously expressed this protein in *Escherichia coli* and biochemically characterized it. ML-005 exhibited lipolytic activity toward short-chained substrates with the preferred substrate being *p*-nitrophenyl-butyrate, suggesting that ML-005 is an esterase. According to homology analysis and site-directed mutagenesis, the catalytic triad of the enzyme was identified as Ser-99, Asp-164, and His-191. Its optimal pH was determined to be at pH 8. Optimal activity was observed at 45°C. It also exhibited temperature, pH and salt tolerance. Residual relative activity after incubating at 50–60°C for 360 min was above 80% of its initial activity. It showed tolerance over a broad range of pH (5–12) and retained most of its initial activity. Furthermore, incubating ML-005 in 1 – 5M NaCl solution had negligible effect on its activity. DTT, EDTA, and ß-mercaptoethanol had no significant effect on ML-005’s activity. However, addition of PMSF led to almost complete inactivation consistent with ML-005 being a serine hydrolase. ML-005 remains stable in the presence of a range of metal ions, but addition of Cu^2+^ significantly reduces its relative activity. Organic solvents have an inhibitory effect on ML-005, but it retained 21% of activity in 10% methanol. SDS had the most pronounced inhibitory effect on ML-005 among all detergents tested and completely inactivated it. Furthermore, the V_max_ of ML-005 was determined to be 59.8 μM/min along with a K_m_ of 137.9 μM. The k_cat_ of ML-005 is 26 s^-1^ and k_cat_/K_m_ is 1.88 × 10^5^ M^-1^ s^-1^.

## Introduction

Lipolytic enzymes have the ability to catalyze the cleavage and synthesis of carboxyl esters (Arpigny and Jaeger, 1999). They are generally classified into two major families according to their substrate specificity: carboxylesterases (3.1.1.1) or “esterases” are active against smaller triglycerides with shorter fatty-acid side chains (<C10), while triacylglycerol hydrolases or “lipases” prefer hydrolyzing water-insoluble triglycerides with longer fatty acid chains (>C10) (Bornscheuer, 2002; Chahiniana and Sarda, 2009; Lopes et al., 2011). Both lipases and esterases show the characteristic α/β hydrolase fold (Ollis et al., 1992). The active site of lipolytic enzymes include a nucleophilic serine residue, a histidine residue and an aspartate or glutamate residue. The serine residue is part of a highly conserved pentapeptide motif called the catalytic elbow, with a consensus motif Gly – X – Ser – X – Gly. In some cases the first glycine residue can be replaced by an alanine resulting in an Ala – X – Ser – X – Gly motif (Dartois et al., 1994; Mala and Takeuchi, 2008). The serine residue is essential for enzyme functionality and lipolytic enzymes are thus part of the “serine-hydrolase” family (Simon and Cravatt, 2010).

Lipolytic enzymes are one of the most important classes of biocatalysts currently in use: they belong to the few enzymes that are produced at an industrial scale exceeding 10^4^ tons per year (Kourist and Gaßmeyer, 2015). According to recent market research, the global enzyme market revenue is expected to reach $ 10.7 billion by 2024 with lipolytic enzymes gaining in importance (Global Market Insights, 2017). They are widely used as industrial catalysts, in the detergent, food and leather industries. Increasing demand in recent years to move away from petrochemical-based industrial processes to more environmentally friendly, bio-based industrial processes is a contributing factor in the increasing interest to utilize lipolytic enzymes in various other industries like pulp and paper, textile, cosmetics industries and biodiesel production (Awaji et al., 1998; Schmid and Verger, 1998; Barron et al., 1999; Mangla et al., 2013; Sharma and Kanwar, 2014). The industrial relevance of lipases is part due to their unique set of versatile characteristics. They are generally resilient against harsh conditions. They can have a broad substrate specificity, but also display chain-length selectivity and enantioselectivity. Lipases and esterases generally do not require any cofactors, they possess a broad pH activity profile, and their relative stability at room temperature in various organic solvents makes them highly attractive for various industries (Gupta et al., 2004; Lotti and Alberghina, 2007). All these factors have led to a renewed research interest in establishing novel enzymatic screening methods, characterizing new enzymes and engineering enzymes with industrially relevant properties.

The search of novel lipolytic enzymes is classically done through functional screening of a multitude of microorganisms for lipolytic activity. This remains a time-proven approach and has led to the discovery of numerous enzymes (Ogawa, 1999). However, this approach can be time-consuming and, more importantly, a majority of the environmental microbes is “non-screenable,” as it is difficult to culture them under laboratory conditions. Rappé and Giovannoni (2003) showed that typically less than 1% of the microbes of an environmental sample grow under “laboratory conditions.” To counter the problem of the uncultivable microbial diversity, total DNA isolated from a microbial community, the so-called “metagenome,” provides an attractive solution (Schloss and Handelsman, 2003). Fragments of this total DNA can be cloned and transformed into desired host systems and the resulting library functionally screened for activity. Due to its unbiased nature, library-based metagenomic analysis led to the discovery of numerous enzymes and continues to be widely used (Henne et al., 2000; Faoro et al., 2012). However, such a screening library typically needs to be massive and optimizing the protein expression-system requires substantial effort, making the screening potentially time-consuming and expensive. Increase in computing power in the past decade has opened up “*in silico* screening” methods. DNA from metagenomic sources is sequenced using “Next Gen” high-sensitivity and high-throughput methods and the resulting sequence database can then be searched for structural motifs of known enzymes by automated bioinformatical analysis (Widmann et al., 2010; Yin et al., 2012). As enzymes are pre-screened *in silico*, only a substantially reduced number of clones need to be biochemically tested for activity, resulting in drastically reduced cost and increased efficiency (Kusnezowa and Leichert, 2017). Rapid advancements in sequencing technologies and drastic reduction in sequencing costs (Goodwin et al., 2016) has led to its widespread adoption in the scientific community. A number of lipolytic enzymes have been identified and biochemically characterized through sequence-based metagenomics (Kwoun Kim et al., 2004; Sharma et al., 2009; Masuch et al., 2015).

Progressing from the above-mentioned DNA-only approaches, we recently described a functional metaproteomics approach, which combines the specificity of activity-based screening with an unbiased meta-omics approach. Through this approach we successfully identified 14 lipolytically active protein spots on a 2D gel derived from the enriched metaproteome of a soil sample harvested from a restaurant’s used cooking oil disposal site. Among those, we identified ML-005, an esterase from a hitherto uncharacterized family, which we could heterologously express in *E. coli* (Sukul et al., 2017).

In the present study, we determine, in detail, the biochemical and enzymatic properties of ML-005, the first lipolytic enzyme found through this functional metaproteomics approach. ML-005 is an esterase with a preference for short-chained substrates. It showed a temperature optimum at 45°C and high stability up to 60°C. It is moderately resistant to organic solvents and detergents, active in neutral to alkaline pH and exhibits high pH- and halotolerance.

## Materials and Methods

### Identification of ML-005

**FIGURE 1:**
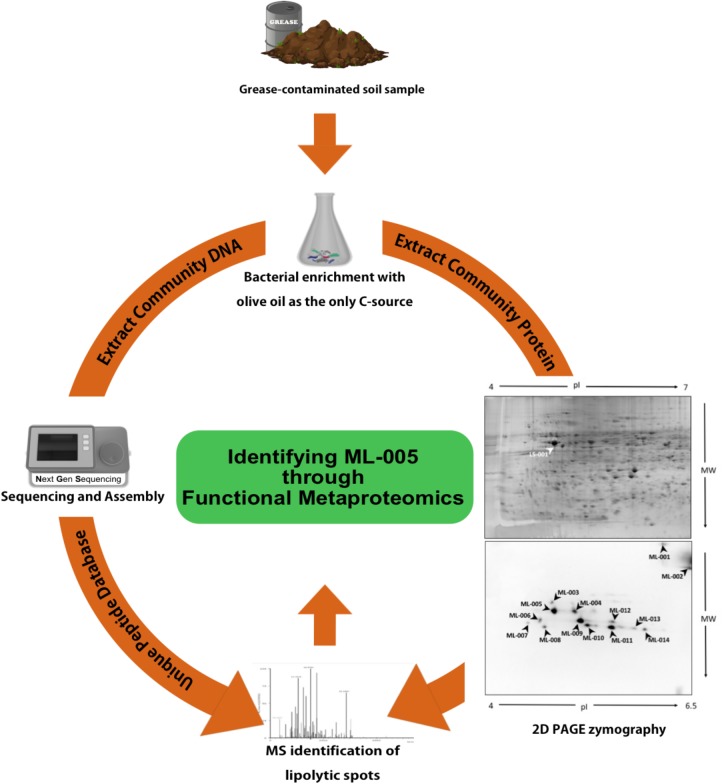
Functional Metaproteomics workflow used to discover ML-005. Grease-contaminated soil sample was used for bacterial enrichment with olive oil as the sole carbon source. Community DNA was extracted, sequenced and annotated. Community protein was separated in a 2D gel and 2D PAGE zymography was carried out to visualize protein spots harboring lipolytic activity. Protein spots were identified using mass-spectrometry, searching against a protein database derived from the metagenomic DNA sequences. ML-005 was selected for this study for further characterization. Functional Metaproteomics has the immediacy of an activity-based approach, while still retaining the comprehensive information of the metagenome (Sukul et al., 2017).

A detailed description of how ML-005 was identified has been published previously (**Figure 1**; Sukul et al., 2017). In short, oil-contaminated soil samples were collected from a restaurant grease disposal site and used for enriching microorganisms with lipolytic activity. Subsequently, the proteins and the DNA contained in the samples were isolated. The proteins were then separated on a 2D gel. In-gel activity assays based on the fluorogenic substrate methylumbelliferyl butyrate enabled us to identify 14 lipolytically active proteins. These proteins were then identified using mass spectrometry by searching against a protein database built from the corresponding metagenomic data. One of the proteins we identified was ML-005, a lipid hydrolase that was hitherto unknown. All metagenomic sequences obtained in the previous study including ML-005’s sequence have been deposited to the European Nucleotide Archive^1^ under project number PRJEB16064 and sample accession number ERP017906. The protein sequence of ML-005 can be found in **Supplementary Data Sheet S2.FASTA**.

### Sequence Analysis of ML-005

Database similarity searches and alignment was performed using BLAST (Marchler-Bauer et al., 2005). The amino acid sequence was analyzed for molecular weight and extinction coefficient using ProtParam on the ExPASy Server (Gasteiger et al., 2005). The three-dimensional structure of ML-005 was predicted using the Phyre^2^ server^2^ (Kelley et al., 2015). The pdb file generated by Phyre was visualized and edited with PyMOL (Schrödinger, LLC, 2015).

### Phylogenetic Analysis and Comparison With Other Microbial Esterases

The sequences of ML-005, Esterases EstA from *Pseudomonas aeruginosa*, Esterase B from *Burkholderia gladioli*, Esterase EstC from *Streptomyces coelicolor*, Esterase EstD from *Thermotoga maritima*, and Carboxylesterase NP from *Bacillus subtilis* were queried against the uniref50 database (release 2018_05, 23-May-2018, Suzek et al., 2015) using the blastp command of the blast command line applications version 2.7.1 (Altschul et al., 1990) with the parameters “-outfmt 10 -evalue 0.0001 -max_target_seqs 100000.” This essentially returned all homologous sequence clusters of these proteins contained in the uniref50 database that form local alignments with an *e*-value below 10^-4^ in a comma separated table file format. The resulting homologs were then ordered in Excel for Mac version 16.14.1 (Microsoft Corporation, Redmond, WA, United States) by “alignment length” and all results with an alignment length below 80% of the length of the original query protein were dismissed. Next the resulting, shortened table was ordered by “% identity” and only results with an identity greater or equal to 35% were considered for further analysis. A batch file was created from these tables containing the uniref50 identifier and the start and end position of the matching alignment in the subject sequence. This batch file was used to retrieve the aligning parts of the subject sequences as a fasta file from the uniref50 database using the blast command line applications’ blastdbcmd command with the “-entry_batch” parameter.

The acquired sequences were imported in Geneious v11.1.4.^3^ (Kearse et al., 2012) and aligned using MUSCLE v*3.8.425* (Edgar, 2004) using default settings. The multiple alignment was used to build a phylogenetic tree using FastTree v2.1.5^4^ (Price et al., 2010) using default settings. FastTree infers approximately maximum-likelihood phylogenetic trees with Jones-Taylor-Thornton (JTT) model of amino acid evolution.

### Heterologous Expression and Purification of ML-005

A detailed description of the heterologous expression und protein purification procedure has been described previously (Sukul et al., 2017). In short, the codon-optimized and artificially synthesized ML-005 gene sequence (645 bp) was cloned into the pET22b expression vector (Merck Millipore, Billerica, MA, United States) containing a T7-promotor. pET22b additionally codes for a C-terminal His_6_-tag that was thus fused to ML-005 (For the vector sequence including insert see **Supplementary Data Sheet S3.FASTA**). ML-005 expression from pET22b in *E. coli* BL21 was induced by the addition of IPTG (1 mM), and was allowed to proceed overnight at 20°C under constant shaking. The cells were harvested by centrifugation (45 min at 6,500 ×*g* and 4°C), were resuspended in in approximately 50 ml of Tris–HCl-buffer (50 mM, pH 7.1), and disrupted by passing the cell suspension through the Constant Cell Disruption System (Constant Systems, Low March, United Kingdom) at 1.9 kbar three times. The resulting lysate was then centrifuged (50 min at 6,500 ×*g* and 4°C) to remove the insoluble fraction from the soluble proteins. The supernatant was then filtered using 250 ml Filtropur V50 vacuum filters (Sarstedt, Nümbrecht, Germany). ML-005 was purified from this soluble protein fraction using an ÄKTApurifier (GE Healthcare, Uppsala, Sweden) with a 5 ml HisTrap HP Ni-NTA-column (GE Healthcare, Uppsala, Sweden). The column was equilibrated using Buffer A (50 mM sodium phosphate, 300 mM NaCl, pH 8.0). The soluble protein fraction was loaded onto the column and ML-005 was eluted with 10–12% Buffer B (50 mM sodium phosphate, 300 mM NaCl, 500 mM imidazole, pH 8.0). The eluate was dialyzed overnight at 4°C against 50 mM sodium phosphate buffer (pH 8) using a Spektra/Por Dialysis Membrane 12–14 kDa (Spectrumlabs, Rancho Dominguez, United States).

### Enzyme Activity Assay

Lipolytic activity of purified ML-005 was determined spectrophotometrically using *p*-nitrophenyl (pNP) esters as substrates. *p*NP-esters are hydrolyzed through lipolytic activity and the released *p*-nitrophenol is yellow and can be detected spectrophotometrically at 405 nm. Unless otherwise described, 38.3 nM (3.92 mg/ml) of purified ML-005 in 50 mM sodium phosphate buffer (pH 8.0) were used to carry out the *p*NP-assays in triplicates. Temperature was varied for different reactions. The hydrolysis reaction was initialized by adding 50 μM of substrate. Reaction volume was 2000 μl. The resulting reaction was followed through a time-course-measurement at 405 nm. One unit (U) of activity was defined as the amount of enzyme releasing 1 μmol of *p*-nitrophenol per minute under the mentioned assay conditions. The extinction coefficient was calculated using a calibration curve. The absorption of different concentrations of *p*-nitrophenol in 50 mM sodium phosphate buffer (pH 8.0) at 25°C was measured at 405 nm.

### Substrate Specificity

Substrate specificity of ML-005 toward pNP-esters with variable acyl chain lengths was measured in 50 mM sodium phosphate buffer (pH 8.0) at 25°C using *p*-nitrophenyl acetate (C2), *p*-nitrophenyl butyrate (C4), *p*-nitrophenyl caprylate (8), *p*-nitrophenyl decanoate (C10), *p*-nitrophenyl laurate (C12), *p*-nitrophenyl myristate (C14), and *p*-nitrophenyl palmitate (C16).

### Temperature Optimum and Temperature Stability

Temperature optimum was determined in the range between 20 and 60°C, in 5°C degree steps, with *p*-nitrophenyl butyrate as the substrate. ML-005 was incubated for 30 min at the necessary temperature and subsequently the activity assay was performed at the same temperature. Temperature-stability of ML-005 was determined for the same temperature points. To determine stability, ML-005 was incubated in the appropriate temperature for 60 and 360 min followed by its residual activity measurement at 25°C and pH 8.

### pH Optimum and pH Stability

pH optimum was determined in the range between 5 and 9.5, in steps of 0.5, with *p*-nitrophenyl butyrate as the substrate. Stability of ML-005 was determined by incubating at pH 4–13 for 360 min followed by the measurement of its residual activity at 25°C and pH 8.

### Halostability

Stability toward high NaCl concentrations was measured by incubating ML-005 at NaCl concentrations of 1 to 5 M. An initial measurement was taken after 3 h and compared with measurements taken after 7 days.

### Effects of Metal Ions, Inhibitors, Detergents, and Organic Solvents on Enzyme Activity

The influence of metal ions was determined using a range of metal salts (CaCl_2_, CuCl_2_, FeCl_2_, KCl, LiCl, MgCl_2_, MnCl_2_, NaCl, and NiCl_2_) at a final concentration of 1 mM.

The effect of inhibitors was determined by using ethylenediaminetetraacetic acid (EDTA), phenylmethylsulfonyl fluoride (PMSF), β-mercaptoethanol (β-ME), and dithiothreitol (DTT) at final concentrations of 1 mM.

Effect of detergents on ML-005 was determined by using sodium dodecyl sulfate (SDS), 3-(3-cholamidopropyl) dimethylammonio-1-propanesulfonate (CHAPS), Tween 20, Tween 80, and Triton X-100 at a final concentration of 1% (v/v) or (w/v) for CHAPS and SDS.

The effect of organic solvents were determined using dimethyl sulfoxide (DMSO), dimethylformamide (DMF), methanol, isopropanol, and acetone at final concentrations of 1 and 10% (v/v).

Control was defined as the reaction mixture in 50 mM sodium phosphate buffer (pH 8.0) without added metal ions, inhibitors, detergents or organic solvents.

Reaction temperature was 25°C and pH was 8.

### Kinetic Parameters

The Michaelis Menten kinetics were investigated with different concentrations (10–600 μM) of *p*NP-butyrate as substrate. The reaction was carried out in 50 mM sodium phosphate buffer (pH 8.0) at the optimum temperature of 45°C. K_m_, k_cat_, and the turnover number was determined, as well as the catalytic efficiency (k_cat_/K_m_). V_max_ and K_m_ were determined through Graphpad Prism using its “Enzyme kinetics – Michaelis-Menten” function. k_cat_ and k_cat_/K_m_ were calculated based on these results.

### Mutagenesis of the Catalytic Triad

Mutations were generated for the catalytic triad residues using the QuikChange method (Stratagene, La Jolla, CA, United States) using the primer pairs listed in **Table 1**. The QuikChange PCR was carried out in a Biometra TProfessional thermal cycler (Whatman Biometra, Goettingen, Germany). 150 ng of plasmid DNA were added to 125 ng each of forward and reverse primer (**Table 1**), 2 mM dNTPs (Thermo Scientific), 2.5 U of Pfu DNA Polymerase (Thermo Scientific), and 5 μl 10 × Pfu DNA Polymerase buffer in a volume of 50 μl. The mutated plasmids were then synthesized by 16 cycles of 30 s 95°C (denaturation), 30 s 58°C (annealing), and 5 min 68°C (elongation), followed by cooling at 4°C until the sample was further processed. The QuikChange PCR product was then digested using DpnI (10 U) for 1 h at 37°C to remove the parental DNA template. Finally the sample was transformed into *E. coli* XL1-Blue cells and the mutagenesis confirmed by sequencing.

**Table 1:** Primers used for QuikChange mutagenesis.

Primer name	Sequence
S99A_f	5′-CGTTGCTCACGCTCTGGGTGTTATCACCCTGCTG-3′
S99A_r	5′-CACCCAGAGCGTGAGCAACGAAGTAGGTGTTTTCG-3′
D164N_f	5′-CTGACAACAACGACCTGGTTCCGCCGAAACTGACC-3′
D164N_r	5′-GCGGAACCAGGTCGTTGTTGTCAGACAGGTAAACCAG-3′
H191N_f	5′-GAACGGTGGTAACTTCCTGGGTCGTGAAGGTTACAC-3′
H191N_r	5′-GACCCAGGAAGTTACCACCGTTCGGAACGGTGATG-3′


Ser-99 was mutated to Ala-99, Asp-164 was mutated to Asn-164, and His-191 was mutated to Asn-191. Following successful mutations, mutated strains (S99A, D164N, and H191N) were cloned into pET22b expression vector and heterologously expressed in *E. coli* BL21. 2 ml of the overexpressed cells were harvested, disrupted through sonication (amplitude: 80%, Cycle: 0.5 s, 3 × 1 min) using a Vial Tweeter (Hielscher, Teltow, Germany). The resulting crude extract was centrifuged (13,000 ×*g*, 20 min, 4°C) to remove cell debris. The supernatant was used for spectrophotometric lipolytic activity determination using pNP-butyrate. Reaction was carried out in 50 mM sodium phosphate buffer (pH 8.0) at 25°C.

## Results

### ML-005 Is a Distant Homolog of the Uncharacterized Esterase YdeN From *Bacillus subtilis*

**FIGURE 2:**
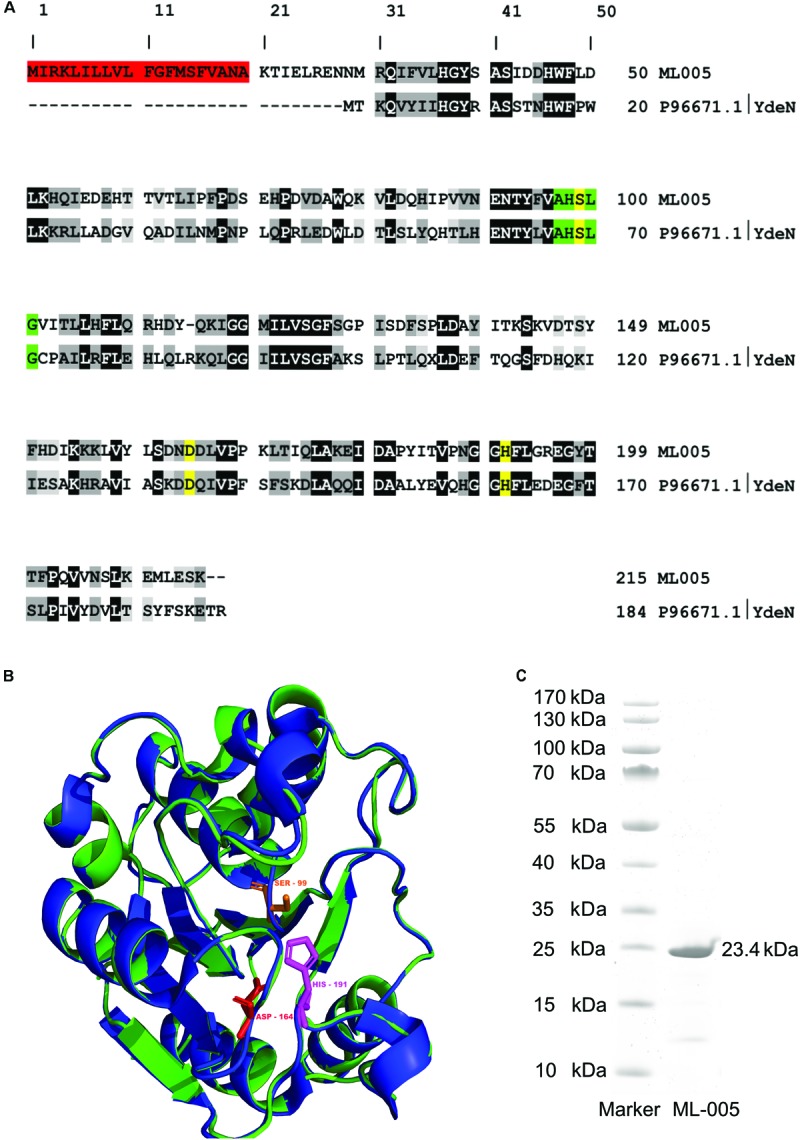
**(A)** Sequence alignment of ML-005 with the uncharacterized esterase YdeN from *Bacillus subtilis* (UniProt accession number P96671.1) showed a sequence identity of 28.44% (highlighted in black, similar amino acids in shades of gray). Residues forming the catalytic triad i.e., serine, (99), aspartic acid (164), and histidine (191) are highlighted in yellow. These residues form an integral part of the active site. The pentapeptide Ala – His – Ser – Leu – Gly motif is highlighted in green. This represents the nucleophilic elbow and is a conserved structure found within lipolytic enzymes. Residues 1–20 form a signal peptide (red) which was processed in *E. coli* (see panel **C**). **(B)** Three dimensional structure of ML-005 was modeled using the Phyre^2^ server (blue ribbon; http://www.sbg.bio.ic.ac.uk/phyre/). Potential catalytic triad residues (His-191, Asp-164, and Ser-99) were predicted to be in close proximity to each other. The structural alignment with YdeN from *B. subtilis* (green ribbon) shows substantial similarity **(C)** ML-005 was cloned into a pET22b vector with a T7 promoter system and a C-terminal His_6_-tag und purified to homogeneity. The purified ML-005 band showed a size that approximates 23.4 kDa, consistent with the calculated mass of His_6_-tagged ML-005 with a removed signal peptide.

A BLASTp search of ML-005 showed it to be a serine hydrolase family protein similar to *B. subtilis* YdeN (UniProtKB – P96671), a predicted esterase with a canonical alpha/beta hydrolase fold (Accession No. COG3545 at NCBI) (Marchler-Bauer et al., 2005; The UniProt Consortium, 2017). The sequence comparison of ML-005 with P96671 showed a sequence identity of 28.44% (**Figure 2A**). Furthermore, a nucleophilic serine residue at position **99** was embedded in a pentapeptide motif composed of Ala-His-Ser-Leu-Gly (97-101). The serine together with aspartic acid residue at position **164** and the histidine residue at position **191** were *in silico* predicted to form the catalytic triad. Three dimensional structure of ML-005 was modeled using the Phyre^2^ server. The catalytic triad residues were found to be in spatial proximity to each other (**Figure 2B**).

The molecular weight of ML-005 was determined to be 24.6 kDa using ProtParam on the ExPASy Server (Gasteiger et al., 2005). With the additional C-terminal His_6_-tag added to the recombinant protein, its theoretical mass was determined to be 25.6 kDa. SignalP (Ferrari et al., 2014) predicted that the initial 20 amino acids are part of a signal peptide sequence (**Supplementary Figure S1**). This hypothesis was confirmed after purification. Purified ML-005 appeared at an apparent size below 25 kDa on an SDS–PAGE, significantly smaller than the predicted 25.6 kDa, indicating the cleavage of the signal-peptide from the newly synthesized protein (see also **Supplementary Figure S2**).

ML-005 was purified to homogeneity (>85%) via a C-terminal His_6_-tag from a pET22b expression vector using *E. coli* BL21 (DE3) as a heterologous host (**Figure 2C**).

### ML-005 Is an Esterase

**FIGURE 3:**
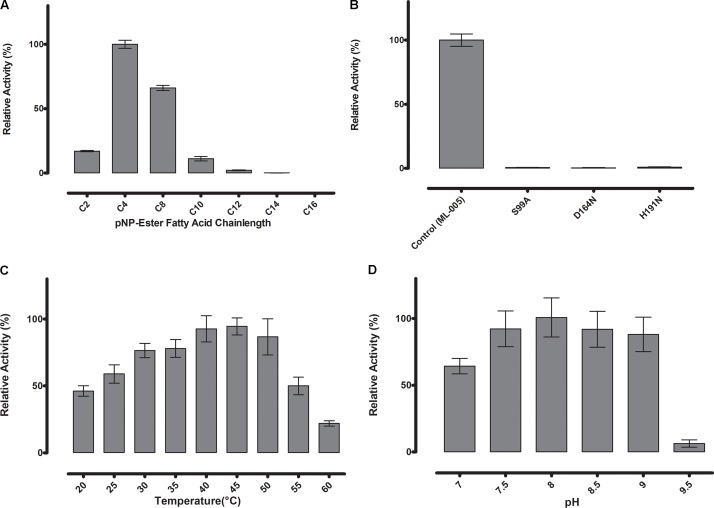
**(A)** Substrate specificity of ML-005 showed clear preference for *p*-nitrophenyl butyrate (C4). Substrate with a chain length of C8 retained approximately 66% of the maximum activity, whereas activity dropped significantly with longer chains. **(B)** Serine (99), aspartic acid (164), and histidine (191) constituting the catalytic triad were mutated and negligible activity was observed for any of the mutants. **(C)** Temperature optimum was observed to be at 45°C. **(D)** pH optimum was observed to be at pH 8, however, activity of ML-005 was largely stable between pH 7.5 and 9. At pH 7, still approximately 64% of maximum activity was retained. pH 9.5 resulted in a drastic loss of activity.

To determine the substrate specificity of ML-005, various chain-lengths of *p*-nitrophenyl esters were tested (C2-C16) (**Figure 3A**). The maximum hydrolysis activity was observed with *p*-nitrophenyl butyrate (C4). An increase in chain-length resulted in lower of activity. pNP-octonoate (C8) showed 66.1% of activity when compared to ML-005’s preferred substrate, pNP-butyrate (14.1 U mg^-1^), while pNP-decanoate (C10), pNP-dodecanoate (C12), and pNP-myristate (C14) retained 11%, 2%, and less than 1%, respectively, while ML-005 showed no detectable enzymatic activity against pNP-palmitate (C16) (**Figure 3A**). This affinity toward short-chained, water-soluble esters classifies ML-005 as a carboxylesterase (EC 3.1.1.1). This classification is consistent with the predicted structure (**Figure 2B**), which lacks a hydrophobic lid, a feature normally present in true lipases and absent from esterases (Brady et al., 1990; Winkler et al., 1990). In contrast, triacylglycerol hydrolase or lipases are generally active against water insoluble long-chained esters (Bornscheuer, 2002). In ML-005 serine (99) along with histidine (191) and glutamate (164) were predicted to be part of the catalytic triad *in silico*. To experimentally confirm the structure-based prediction of the catalytic triad, we mutagenized these three residues. As expected, all three mutants S99A, D164N and H191N showed negligible activity (<1%) compared to wild type, thus experimentally confirming ML-005 to be a classic serine hydrolase (**Figure 3B**).

### ML-005 Is a Member of an Underexplored Family of Microbial Esterases

**FIGURE 4:**
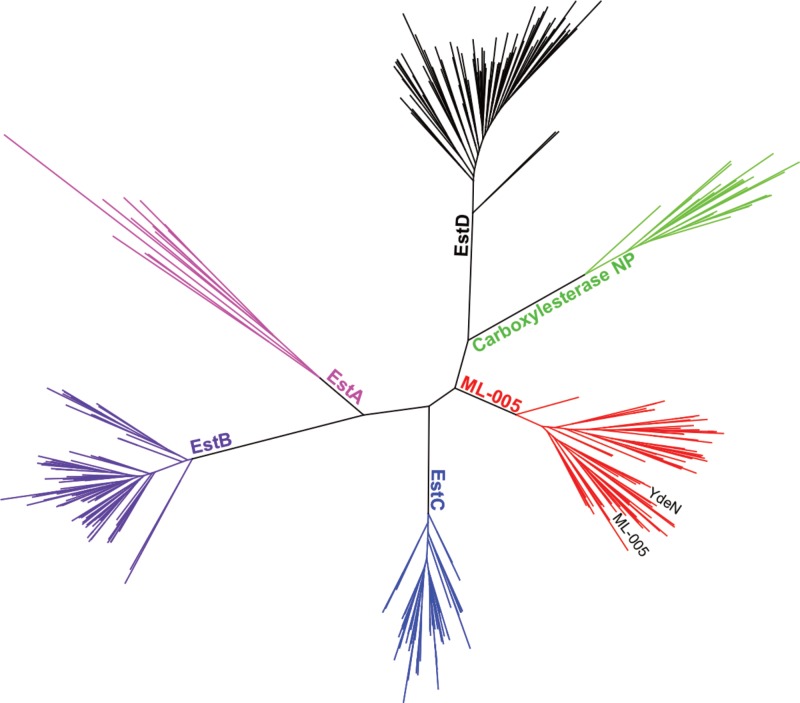
Phylogenetic comparison of ML-005 and 5 other well-characterized bacterial esterases. Tree of lipase sequences with 35% identity to ML-005 and to esterases EstA from *P. aeruginosa*, EstB from *B. gladioli*, EstC from *S. coelicolor*, EstD from *T. maritima* and Carboxylesterase NP from *B. subtilis*. ML-005 is member of an as of yet uncharacterized family of esterases, which also contains YdeN from *B. subtilis*.

In order to justify an in-depth characterization of ML-005’s biochemical properties, we compared ML-005 to 5 other microbial esterases that have been well characterized. These included Esterases EstA from *P. aeruginosa* (Wilhelm et al., 1999), Esterase EstB from *B. gladioli* (Wagner et al., 2002), Esterase EstC from *S. coelicolor* (Brault et al., 2012), Esterase EstD from *T. maritima* (Levisson et al., 2007), and Carboxylesterase NP from *B. subtilis* (Quax and Broekhuizen, 1994). A phylogenetic tree revealed that ML-005 formed a distinct branch with 70 clusters representing 825 proteins found in the uniref50 database (**Figure 4**). To our knowledge, none of these proteins have been characterized so far. We therefore decided to biochemically characterize this metagenomic enzyme as the first representative of this novel family in more detail.

### ML-005 Has a Mesophilic to Thermophilic Temperature Preference and Retains Maximum Activity in a Range of pH 7.5 to 9

Thermostability and pH-optimum are critical parameters when choosing biocatalysts. We therefore measured the hydrolytic activity of ML-005 at temperatures ranging from 20 to 60°C. The optimum temperature for ML-005 was determined to be 45°C (**Figure 3C**). At 55°C, it still retained 50% of its activity and approximately 22% activity was retained at 60°C. This suggests a slightly thermophilic activity profile. Purified ML-005 exhibited high activity between pH 7.5 and pH 9 with pH 8 being the optimum pH with the highest activity (**Figure 3D**). At pH 7, ML-005 had 64% of maximum activity while pH 9.5 resulted in a drastic breakdown in activity with only 6% of maximum activity remaining.

### ML-005 Is Tolerant of High Temperature, High pH and High NaCl Concentrations

**FIGURE 5:**
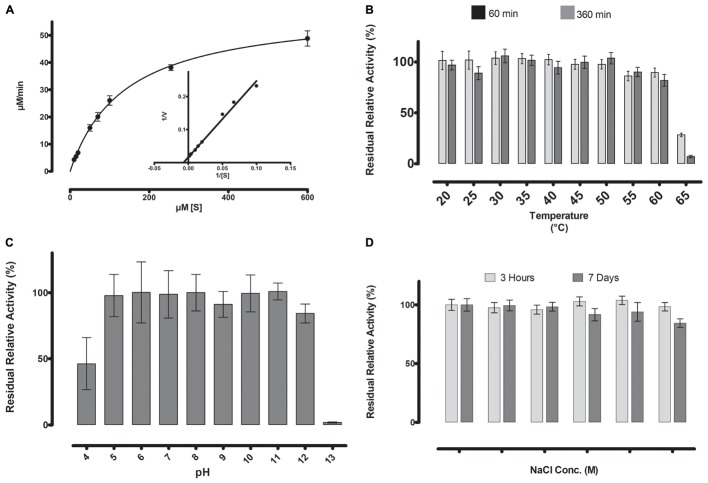
**(A)** Michaelis-Menten kinetics were observed for ML-005 with pNP-butyrate. V_max_ of ML-005 was determined to be 59.8 μM/min along with a K_m_ of 137.9 μM. The k_cat_ of ML-005 is 26 s^-1^ and k_cat_/K_m_ is 1.88 × 10^5^ M^-1^ s^-1^. **(B)** ML-005 showed temperature tolerance from 20 to 60°C. **(C)** ML-005 showed tolerance over a broad range of pH (5–12) and retained most of its activity. At pH 4 it retained approximately 50% of its activity while pH 13 almost completely deactivated ML-005. **(D)** ML-005 showed halotolerance when incubated in increasing NaCl concentrations. After 7 days of incubation at close to saturated NaCl solution (5M), ML-005 still retained most of its activity.

The stability of ML-005 was determined by measuring the residual activity with *p*-nitrophenyl-butyrate (C4) at optimum conditions after incubation at a wide range of temperature, pH and NaCl concentration. After incubation at 60°C for 360 min ML-005 still retained an activity of 80% when compared to optimum conditions (**Figure 5B**). ML-005 retained activity close to its optimum activity when incubated between pH 5 to pH 12 for 360 min. Incubating ML-005 at pH 4 led to a decrease in activity to approximately 46%, while on the basic side, incubating ML-005 at pH 13 resulted in the loss of most of its activity (2%) (**Figure 5C**). Incubating ML-005 at 1 to 5 M of NaCl concentration, even for 7 days, showed only negligible effect on its activity (**Figure 5D**), indicating that ML-005 is a halotolerant enzyme.

### Enzymatic Parameters of ML-005

With the optimal conditions at hand, we determined ML-005’s Michaelis-Menten kinetics by measuring its activity at various substrate concentrations at pH 8 and 45°C (**Figure 5A**). The V_max_ of ML-005 was determined to be 59.8 μM/min and the K_m_ was determined to be 137.9 μM. The k_cat_ of ML-005 is 26 s^-1^ and its catalytic efficiency k_cat_/K_m_ is 1.88 × 10^5^ M^-1^ s^-1^.

### Serine Is an Integral Part of the Active Site of ML-005

**FIGURE 6:**
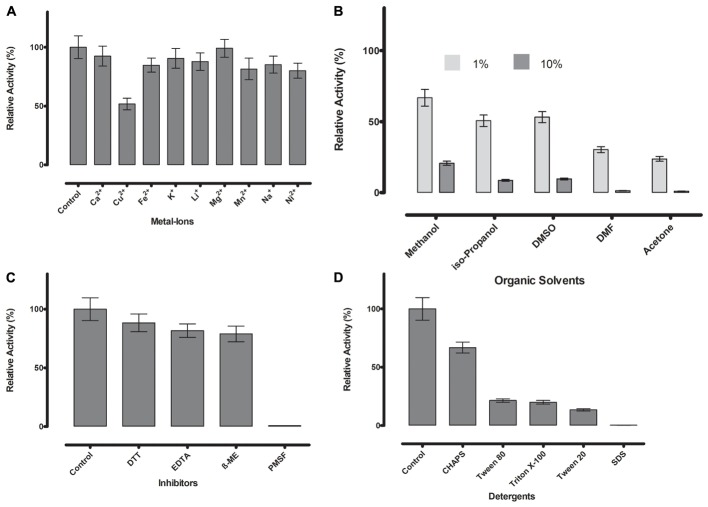
**(A)** Metal ions showed negligible effect on ML-005 at a concentration of 1 mM, with copper showing the most drastic effect by inhibiting ML-005 by approximately 50%. **(B)** Organic solvents had an overall inhibiting effect on ML-005 without exception, but ML-005 was moderately stable in the presence of even 10% Methanol. **(C)** All inhibitors showed moderate inhibiting effect at a concentration of 1 mM, with relative activity staying at around 80%. Only PMSF showed almost complete inhibition of ML-005, consistent with an active-site serine. **(D)** Detergents at 1% were also found to have an overall inhibiting effect. CHAPS showed the least effect with 66% relative activity and SDS inactivating ML-005 completely with negligible remaining activity.

To further determine ML-005’s resilience, the modulating or inhibiting effects of various compounds on its activity were studied (**Figure 6**).

Divalent cations are known to have inhibitory or stimulating effect on lipase and esterase activity (Gupta et al., 2004). Most are considered to have an inhibitory effect, but the majority of metal ions we tested (Ca^2+^, Fe^2+^, K^+^, Li^+^, Mg^2+^, Mn^2+^, Na^+^, and Ni^2+^) showed no or negligible effects at 1 mM concentration in our assay (**Figure 6A**). Only Cu^2+^ reduced the activity substantially to 52%, a known phenomenon with lipolytic enzymes (Hiol et al., 2000).

Resilience against organic solvents is of particular interest for industrial applications, as esterases are widely used in processes like the synthesis of pharmaceutical and fine chemicals where these solvents are present (Fuciños et al., 2012). All tested organic solvents showed an inhibiting effect on ML-005. 1% of acetone, DMF, DMSO, isopropanol and methanol diminished the activity to approximately 24, 30, 53, 51, and 67% of the original activity, respectively. 10% organic solvent diminished ML-005 activity even more, nevertheless ML-005 still retained 21% of its activity in 10% Methanol (**Figure 6B**), making it potentially usable in applications such as methyl-esterification of organic substrates.

Presence of DTT, EDTA and ß-mercaptoethanol at a concentration of 1 mM had a slight inhibiting effect on ML-005 with 88, 81, and 79% of remaining activity compared to control, respectively. However, based on these results we conclude that neither structural disulfides or coordinated metals play a role in ML-005’s activity (**Figure 6C**). PMSF inactivated ML-005 completely with only 0.7% residual relative activity. PMSF is most likely acting as a specific inhibitor and irreversibly deactivates ML-005 by binding to the nucleophilic serine of the active site (Narayanan and Jones, 2015). This further confirmed to us that, unlike some lipases, ML-005 cannot resist the specific inhibition of PMSF by physically hindering access to the active site with the help of a hydrophobic lid (Côté and Shareck, 2008).

Lastly we tested the effect of detergents on ML-005 (**Figure 6D**). All detergents showed inhibition with CHAPS, SDS, Tween 20, Tween 80, and Triton X-100 showing a relative activity of 66, 0.2, 13, 21, and 19%, respectively, at a 1% concentration. ML-005 showed the most resilience against CHAPS and was most susceptible to the effects of SDS.

## Discussion

ML-005 was identified in a previous study through a functional metaproteomics approach. In this preceding study, enrichment cultures from grease-contaminated soil samples were used to grow oil-metabolizing bacteria. The metaproteome acquired from the enrichment cultures was then screened for lipolytic activity. The metagenome was simultaneously extracted from the same biological samples followed by its sequencing and annotation. This enabled us to excise ML-005 based on its lipolytic activity and identify it through MS by searching against a protein database derived from the metagenome (Sukul et al., 2017). In the present study we carry out a comprehensive characterization of this novel esterase ML-005.

Sequence analysis revealed that ML-005 is a distant homolog of YdeN from *B. subtilis*. YdeN is a canonical alpha/beta hydrolase, which is part of a family of esterases that to our knowledge has not been biochemically characterized yet. To our knowledge, characterization of ML-005 is the first characterization of a member of this novel family and could help provide information about the characteristics of this family of lipolytic enzymes. The crystal structure of *B. subtilis* YdeN has been resolved previously (pdb – 1UXO) and provided insights over its putative functionality. The presence of a complete catalytic triad indicated hydrolytic activity but the absence of a hydrophobic lid suggested that it is possibly active against water-soluble esters or various thioesters (Janda et al., 2004). In the case of ML-005, a nucleophilic serine residue embedded in the characteristic pentapeptide motif composed of Ala-His-Ser-Leu-Gly strongly suggested hydrolytic activity. While non-catalytic alpha-beta hydrolases have been previously identified, these typically lacked the nucleophilic serine (Wilson et al., 2004). The pentapeptide in ML-005 is modified from the more common Gly-X-Ser-X-Gly, a highly conserved sequence feature of lipolytic enzymes (Mala and Takeuchi, 2008). Such a modified pentapeptide is typically found in thermostable lipases from *Bacillus* spp. and *Geobacillus* spp. (Cho et al., 2000; Masomian et al., 2016). ML-005 has a mesophilic to thermophilic temperature preference. It showed a relatively high optimum temperature at 45°C while still retaining 50% of its activity at 55°C. This is in contrast to thermostable lipases from *Bacillus* spp. and *Geobacillus* spp. where the optimum temperature range is between 55 and 70°C (Masomian et al., 2016). ID-1 from *Bacillus thermoleovorans* showed even higher optimal temperature at 75°C. On the other hand ML-005 showed higher temperature stability. ID-1 retained 50% of its activity after exposure at 60°C for 60 min, whereas ML-005 retained approximately 90% of its activity after 60 min and approximately 80% of its activity after 360 min (Cho et al., 2000). In addition to its elevated temperature profile, ML-005 is active in neutral to alkaline pH. This is similar to esterases EstOF4 and Carboest; both from *Bacillus spec* (Karpushova et al., 2004; Rao et al., 2013). At pH 9.5 ML-005 is reversibly inactivated, but it did show a substantially broad pH-tolerance and regained close to its full activity in optimal buffer conditions even after long incubation at pH 5 on the acidic side and pH 12 on the basic side. This is in contrast to multiple esterases characterized, where the tolerances were confined to a narrower pH range (Alex et al., 2014; Kang et al., 2017).

For lipolytic enzymes, substrate specificity may be used to distinguish between carboxylesterases and lipases (Lopes et al., 2011). Numerous carboxylesterases, active against water-soluble short-chained esters, have been previously characterized. Esterases like Ly-2 from *Brevundimonas* or B2^T^ from *Pelagibacterium* showed their preference for C2/C4 sidechains while Rv0045c from *Mycobacterium* showed preference for a C6 substrate and even others like EstMY isolated from metagenomic origin showed preference for a C8 substrate (Guo et al., 2010; JunGang et al., 2010; Jiang et al., 2012; Zhang et al., 2017). This is in contrast to lipases which are active against long-chained esters. For example, *Acinetobacter* LipA or SRT-9 from *Pseudomonas aeruginosa* showed optimal activity for longer sidechains ranging from C10-C18 (Kok et al., 1995; Borkar et al., 2009; Kumar et al., 2016). ML-005 is an esterase with a preference for short-chained substrates. It shows its maximum activity toward pNP-C4 with its activity decreasing with increasing chain length. This bears similarity to other esterases found previously like est_p1 (Peng et al., 2011).

We also determined the kinetic properties of ML-005 against *p*NP-butyrate. With a k_cat_ of 26 s^-1^, and a K_M_ of 137.9 μM and a catalytic efficiency of k_cat_/K_m_ of 1.88 × 10^5^ M^-1^ s^-1^, ML-005 has enzymatic parameters fairly typical for esterases already characterized over the years. FNE, lipG, HydS14, and AT4 showed lower catalytic efficiency than ML-005, with HydS14 showing a catalytic efficiency as low as 1.17 × 10^4^ M^-1^ s^-1^ (Lee et al., 2006; Park et al., 2006; Yu et al., 2010; Sriyapai et al., 2015). EstI, EStEP16, and PDF1Est showed better catalytic properties than ML-005 with EStEP16 showing an efficiency of 1.58 × 10^6^ M^-1^ s^-1^ (Choi et al., 2004; Ay et al., 2011; Zhu et al., 2013).

ML-005 is a serine hydrolase with the nucleophilic serine integrally embedded in the active site. 3D-structure of ML-005 was modeled using Phyre^2^ and Ser_99_, Asp_164_, and His_191_ were found to be in close proximity to each other. These residues coordinate together to form a charge-relay-network at the active site and are key to the functionality of esterases and lipases (Brady et al., 1990; Dodson and Wlodawer, 1998). Consequently, our mutagenesis of these three amino acid residues resulted in a virtually complete loss of activity. Furthermore, PMSF, a serine inhibitor, deactivates ML-005. While there are known lipases and esterases that are resistant to PMSF (Makhzoum et al., 1996; Abramić et al., 1999; De Santi et al., 2016), a majority of esterases do not sterically hinder the PMSF to access the active site and are as such sensitive against it (Xin and Ying, 2013; Zhu et al., 2015; Bakir and Metin, 2016; Samoylova et al., 2018).

Mg^2+^, Ca^2+^, and Na^+^ have been shown to have a stimulating effect on lipolytic enzymes (Rathi et al., 2001; Sharma et al., 2002), however, we did not observe a significant effect with these metals. Cu^2+^ at 1 mM reduced the activity substantially to 51.71%, this phenomenon has been previously observed (Hiol et al., 2000).

In general terms, all tested organic solvents showed substantial inhibitory effect on ML-005. Only with 1% methanol, ML-005 showed 67% remaining activity and even with 10% methanol, 21% of activity remained. While there are a number of known solvent-resistant lipolytic enzymes, there are many that are unstable in organic solvents (Doukyu and Ogino, 2010). Protein engineering provides an interesting solution to the problem. For *Geobacillus stearothermophilus* lipase T6, two protein engineering approaches, random mutagenesis and structure-guided consensus provided mutants that showed stability improvement of 23-fold and 66-fold, respectively, with elevated half-life in 70% methanol making it viable for biodiesel production (Dror et al., 2014). Directed evolution was also used for producing the methanol-tolerant Dieselzyme 4, which outperforms the industrially used lipase from *Burkholderia cepacia* for biodiesel production (Korman et al., 2013). Protein engineering approaches may even be used to change substrate specificity in specific organic solvents. In the case of the well-known industrial lipase *Candida antarctica* Lipase B (CALB), a point mutation in the stereospecificity pocket resulted in 270-fold increase of the specificity constant for the acylation of bulky, non-native substrates in cyclohexane, when compared to wildtype (Hudson et al., 2005).

All detergents showed inhibitory effect while SDS resulted in a drastic loss of activity. Detergents have not only consequences for enzyme-detergent interaction but also for enzyme-substrate interaction. Activity can be promoted in some lipases where surfactant binding results in an increase of activity due to greater availability of substrate and increased access to the active site (Delorme et al., 2011) Exposure to SDS, an anionic detergent, may lead to tertiary conformational changes and thus may result in loss of activity (Rao et al., 2013).

Over the last decade, significant steps are being taken to transition from a petrochemical-based economy toward a sustainable bio-based economy. Biocatalysis is a necessary piece of the puzzle and searching for novel enzymes with novel characteristics continue to be worthwhile. Intrinsically stable enzymes provide a promising starting point for protein engineering endeavors to evolve enzymes that can be used for highly specialized industrial applications (Bornscheuer et al., 2012; Bornscheuer and Kourist, 2017). ML-005 is a robust esterase that is tolerant toward elevated temperature and a broad range of pH and may provide an interesting starting point for directed evolution.

## Data Availability Statement

The raw data supporting the conclusions of this manuscript will be made available by the authors, without undue reservation, to any qualified researcher. All strains used in this study are available upon request from the authors.

## Author Contributions

PS and LL designed the experiments. NL and PS purified ML-005. PS performed the enzymatic characterization of ML-005. PS and LL wrote the manuscript.

## Conflict of Interest Statement

The authors declare that the research was conducted in the absence of any commercial or financial relationships that could be construed as a potential conflict of interest.

## Footnotes

1 www.ebi.ac.uk/ena

2 http://www.sbg.bio.ic.ac.uk/phyre/

3 http://www.geneious.com

4 http://www.microbesonline.org/fasttree

## Supplementary Material

FIGURE S1 SignalP prediction server predicted the presence of a signal peptide with a cleavage site between position 20 and 21 (Ferrari et al., 2014).

FIGURE S2 Alignment of ML-005 with and without signal sequence and comparison with *Bacillus subtilis* YdeN (P96671).

DATA SHEET S2.FASTA Supplementary File 1: Protein sequence of ML-005.

DATA SHEET S3.FASTA Supplementary File 2: DNA sequence of pML-005.
