# Corrigendum: Characterization of ML-005, a Novel Metaproteomics-Derived Esterase

**DOI:** 10.3389/fmicb.2018.02716

**Published:** 2018-11-14

**Authors:** Premankur Sukul, Natalie Lupilov, Lars I. Leichert

**Affiliations:** Department of Microbial Biochemistry, Institute of Biochemistry and Pathobiochemistry, Ruhr University Bochum, Bochum, Germany

**Keywords:** esterase, lipase, metagenomics, metaproteomics, biocatalysis

In the original article, there was a mistake in Figures 5 and 6 as published. These two figures were inadvertently swapped. The corrected Figures 5, 6 appear below.

**Figure 5:**
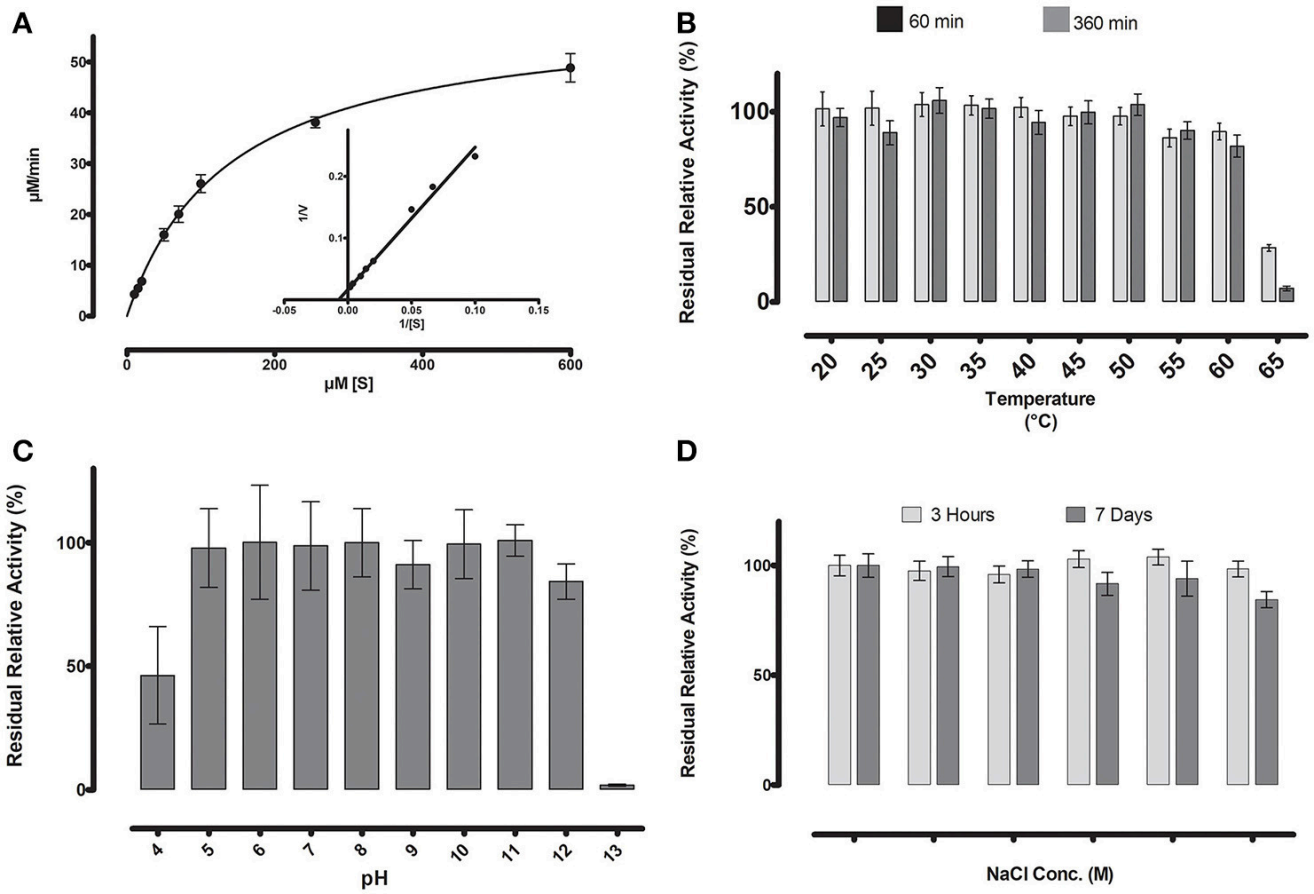
**(A)** Michaelis-Menten kinetics were observed for ML-005 with pNP-butyrate. V_max_of ML-005 was determined to be 59.8 μM/min along with a K_m_ of 137.9 μM. The k_cat_ of ML-005 is 26 s^−1^and k_cat_/K_m_ is 1.88 × 10^5^ M^−1^ s^−1^. **(B)** ML-005 showed temperature tolerance from 20 to 60°C. **(C)** ML-005 showed tolerance over a broad range of pH (5–12) and retained most of its activity. At pH 4 it retained ~50% of its activity while pH 13 almost completely deactivated ML-005. **(D)** ML-005 showed halotolerance when incubated in increasing NaCl concentrations. After 7 days of incubation at close to saturated NaCl solution (5M), ML-005 still retained most of its activity.

**Figure 6:**
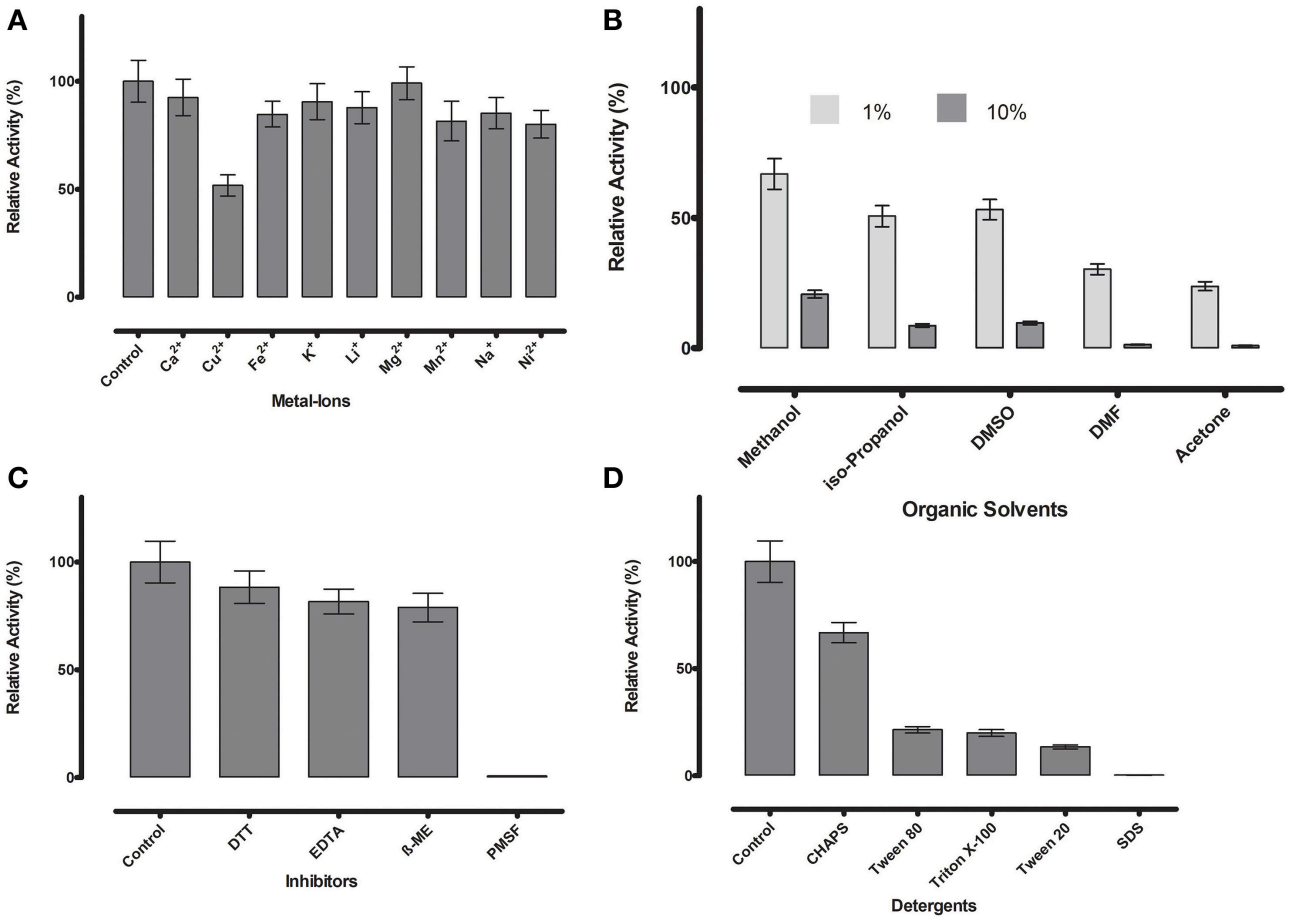
**(A)** Metal ions showed negligible effect on ML-005 at a concentration of 1 mM, with copper showing the most drastic effect by inhibiting ML-005 by ~50%. **(B)** Organic solvents had an overall inhibiting effect on ML-005 without exception, but ML-005 was moderately stable in the presence of even 10% Methanol. **(C)** All inhibitors showed moderate inhibiting effect at a concentration of 1 mM, with relative activity staying at around 80%. Only PMSF showed almost complete inhibition of ML-005, consistent with an active-site serine. **(D)** Detergents at 1% were also found to have an overall inhibiting effect. CHAPS showed the least effect with 66% relative activity and SDS inactivating ML-005 completely with negligible remaining activity.

The authors apologize for this error and state that this does not change the scientific conclusions of the article in any way. We thank Ruth Nahurira for bringing this to our attention. The original article has been updated.

## Conflict of interest statement

The authors declare that the research was conducted in the absence of any commercial or financial relationships that could be construed as a potential conflict of interest.

