# Supplementary material for: Characterization of ML-005, a Novel Metaproteomics-Derived Esterase
Source: Front Microbiol. 2018 Aug 22;9:1925. doi: 10.3389/fmicb.2018.01925 (PMC6119806; doi:10.3389/fmicb.2018.01925)
Supplement: FIGURE S1 [file Image_1.pdf]

frontiers

Supplementary Material

Characterization of ML-005, a Novel Metaproteomics-Derived Esterase

Premankur Sukul[1], Natalie Lupilov[1], Lars I. Leichert[1]*

[1]Department of Microbial Biochemistry, Institute of Biochemistry and Pathobiochemistry, Ruhr University Bochum, Bochum, Germany

*** Correspondence:**
Lars I. Leichert
lars.leichert@ruhr-uni-bochum.de

Supplementary Figures

```
# SignalP-4.1 euk predictions
>Sequence
```

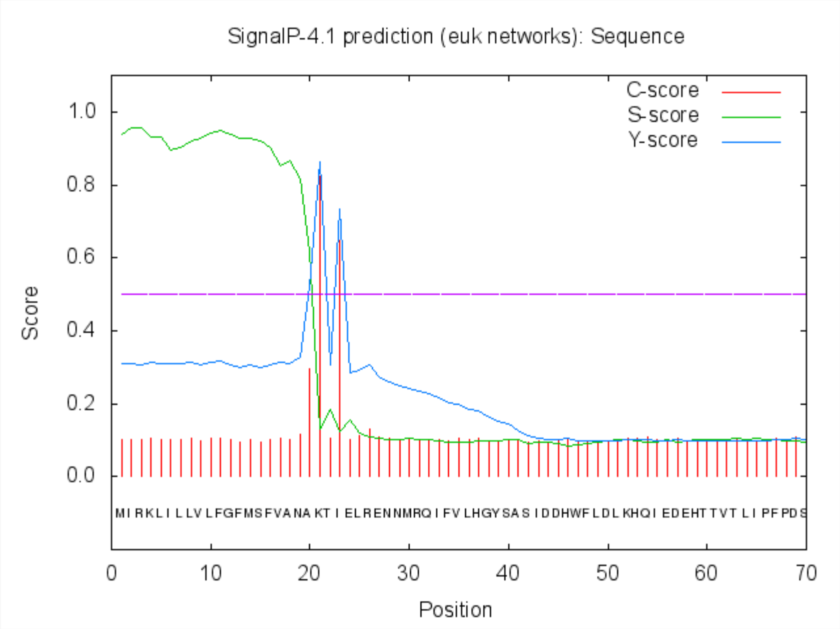


```
# Measure  Position  Value    Cutoff   signal peptide?
  max. C    21       0.824
  max. Y    21       0.863
  max. S     3       0.958
  mean S     1-20    0.901
       D     1-20    0.884   0.450    YES
Name=Sequence   SP='YES' Cleavage site between pos. 20 and 21: ANA-KT D=0.884 D-cutoff=0.450 Networks=SignalP-noTM
```

Supplementary Figure S1: SignalP prediction server predicted the presence of a signal peptide with a cleavage site between position 20 and 21 (Ferrari et al., 2014)

```
CLUSTAL O(1.2.4) multiple sequence alignment

ML-005_without_SignalSeq      --------------------KTIELRENNMRQIFVLHGYSASIDDHWFLDLKHQIEDEHT
ML005_with_SignalSeq          MIRKLILLVLFGFMSFVANAKTIELRENNMRQIFVLHGYSASIDDHWFLDLKHQIEDEHT
YDEN_P96671                   ----------------------------MTKQVYIIHGYRASSTNHWFPWLKKRLLADGV
                                                            :*::::*** **  :***  **:::  : .

ML-005_without_SignalSeq      TVTLIPFPDSEHPDVDAWQKVLDQHIPVVNENTYFVAHSLGVITLLHFLQRHDY-QKIGG
ML005_with_SignalSeq          TVTLIPFPDSEHPDVDAWQKVLDQHIPVVNENTYFVAHSLGVITLLHFLQRHDY-QKIGG
YDEN_P96671                   QADILNMPNPLQPRLEDWLDTLSLYQHTLHENTYLVAHSLGCPAILRFLEHLQLRKQLGG
                               . :: :*:  :* :: * ..*. :  .::****:******  ::*:**:: :  :::**

ML-005_without_SignalSeq      MILVSGFSGPISDFSPLDAYITKSKVDTSYFHDIKKKLVYLSDNDDLVPPKLTIQLAKEI
ML005_with_SignalSeq          MILVSGFSGPISDFSPLDAYITKSKVDTSYFHDIKKKLVYLSDNDDLVPPKLTIQLAKEI
YDEN_P96671                   IILVSGFAKSLPTLQMLDEFTQGSFDHQKIIESAKHRAVIASKDDQIVPFSFSKDLAQQI
                              :******:  :  :. ** :   *  . . :.. *:: *  *.:*::** .:: :**::*

ML-005_without_SignalSeq      DAPYITVPNGGHFLGREGYTTFPQVVNSLKEMLESKLEHHHHHH
ML005_with_SignalSeq          DAPYITVPNGGHFLGREGYTTFPQVVNSLKEMLESKLEHHHHHH
YDEN_P96671                   DAALYEVQHGGHFLEDEGFTSLPIVYDVLTSYFSKETR------
                              **    * :*****  **:*::* * : *.. :..: .
```

Supplementary Figure S2: Alignment of ML-005 with and without signal sequence and comparison with *Bacillus subtilis* YdeN (P96671).
